# Effects of auditory rhythmic adaptation on lower limb joint mechanics during single-leg drop landings in individuals with functional ankle instability

**DOI:** 10.3389/fnhum.2025.1579260

**Published:** 2025-07-02

**Authors:** Lingyue Meng, Yubo Wang, Zilong Wang, Yongan Liu, Yong Tan, Yue Zhang, Xinhui Wei, Xiaokun Mao, Qiuxia Zhang

**Affiliations:** ^1^Physical Education and Sports School, Soochow University, Suzhou, Jiangsu, China; ^2^Rehabilitation Engineering Lab, Department of Kinesiology and Community Health, University of Illinois at Urbana-Champaign, Champaign, IL, United States

**Keywords:** functional ankle instability, drop landing, auditory, rhythmic adaptation, joint mechanics

## Abstract

**Objective:**

This study investigates the effects of auditory rhythmic adaptation on lower limb joint mechanics in individuals with Functional Ankle Instability (FAI) during drop landings, aiming to explore potential rehabilitation strategies.

**Methods:**

Twenty male FAI individuals performed single-leg drop landings under four rhythmic conditions (no rhythm, 60, 120, 180 bpm) after auditory rhythmic adaptation. Joint mechanics data were collected, and analyzed using two-way repeated measures ANOVA to examine the main effects and interaction effects of rhythm and limb condition. Rhythmic adaptation was assessed using time interval reproduction paradigm.

**Results:**

The ground reaction force (GRF), joint torque and joint stiffness were significantly influenced by side (*p*< 0.05). Hip and knee joint range of motion (RoM), lower limb and joint stiffness, joint torque were significantly affected by conditions (*p*< 0.05). Significant interaction effects were observed in joint stiffness and joint torque (*p* < 0.05).

**Conclusion:**

Rhythmic auditory adaptation modulates motor control strategies in individuals with FAI by influencing joint mechanics during drop landing. In particular, rhythmic adaptation at 120 bpm facilitates a proximal-dominant torque-redistribution strategy, characterized by higher hip and knee extension torques and increased ankle plantarflexion torque on the stable side, and increased hip extension torques on the stable side. These changes suggest the potential of 120 bpm to improve motor control and reduce injury risk.

## 1 Introduction

Drop landing is a common movement in both daily activities and sports, and the associated ground reaction forces (GRF) can reach several times body weight (Puddle and Maulder, 2013; Saunders et al., 2014), posing a high risk of ankle injuries. Due to its unique anatomical structure, the ankle is particularly prone to plantar flexion and inversion during drop landing, increasing the likelihood of lateral ligaments damage (Fong et al., 2009). Fransz et al. (2018) identified the late dynamic component of GRF during single-leg drop landing as a significant predictor of non-contact lateral ankle sprains, underscoring the close link between altered landing mechanics and injury risk. Therefore, optimizing joint mechanics seems to be critical for reducing the risk of ankle injuries during drop landings.

However, during actual athletic performance, ankle injuries are often underestimated or inadequately managed. Research has shown that more than half of athletes (56.8%) who suffer ankle injuries do not seek professional treatment (McKay et al., 2001), opting instead for self-rehabilitation. Additionally, unresolved post-injury inflammation and inadequate rehabilitation following ankle sprains can disrupt neuromuscular control, leading to movement pattern alterations (Hertel, 2002). These maladaptive changes often involve altered joint kinematics and delayed muscle activation, which persist even after acute symptoms resolve (Marín Fermín et al., 2023). As a consequence of an initial ankle sprain, approximately 40% of individuals go on to develop chronic ankle instability (CAI) (Hertel, 2002). CAI serves as an overarching term that encompasses a range of post-sprain dysfunction and can be categorized into mechanical ankle instability (MAI), which involves structural laxity, and functional ankle instability (FAI), which occurs in the absence of such mechanical deficits but is associated with impaired neuromuscular control(Freeman, 1965). While aging (Konrad et al., 1999) and fatigue (Edwards, 1981) independently contribute to movement dysfunction, the presence of unresolved ankle injuries further amplifies these risks. Over time, FAI individuals may develop compensatory movement patterns as a protective mechanism, which can result in diminished motor control, delayed muscle response time, neuromuscular dysfunction, and lower limb asymmetry (Monteleone et al., 2014). These issues, in turn, may impair the ability of central nervous system (CNS) to integrate and process sensory information (Cyr et al., 2019; Henry and Baudry, 2019).

Proprioceptive dysfunction is considered a hallmark feature of FAI that may contribute to postural instability and inefficient motor strategies (Proske and Gandevia, 2012). Since effective motor control depends on the integration of multiple sensory inputs, including proprioceptive, visual, and auditory information, the disruption of proprioception may lead individuals with FAI to rely more heavily on alternative sensory modalities to maintain postural control and functional performance (Meng et al., 2022). Among these modalities, auditory input has gained increasing attention for its role in enhancing movement coordination. Rhythmic auditory stimulation (RAS), which provides external timing cues through rhythmic sounds, has been shown to facilitate motor control by promoting auditory-motor synchronization in both healthy individuals and clinical populations (Braun Janzen et al., 2021). RAS has been demonstrated to modulate spatiotemporal gait parameters, improve joint kinematics, and enhance motor cortical excitability (Thaut et al., 2019) by engaging cerebellar and subcortical structures involved in timing and coordination (Shin et al., 2015). Based on the neural entrainment framework, RAS provides a temporally structured cueing system that supports anticipatory postural adjustments and improves movement efficiency. This is particularly relevant for individuals with FAI, who often exhibit an earlier peak in vertical GRF and a delay in the activation of musculoskeletal components during drop landing(Jeon et al., 2021), indicating deficits in motor timing and preparatory control. RAS may therefore serve as an effective compensatory mechanism, reinforcing temporal structure in motor execution and mitigating neuromuscular delays associated with proprioceptive deficits.

Preliminary research has revealed the potential of auditory rhythm in improving balance in individuals with ankle instability (Coker et al., 2023). Therefore, auditory rhythmic cues may hold considerable promise in the rehabilitation of individuals with FAI, warranting further exploration to clarify their underlying mechanisms and therapeutic value. We hypothesized that auditory rhythmic cues would modulate lower limb joint range of motion and torques, and that different rhythms would elicit distinct bilateral limb responses during drop landing. Accordingly, our goal was to explore the status of the lower limb joint mechanics among FAI individuals, to identify which outcomes displayed deficits and which outcomes were sensitive to detect these different rhythmic conditions.

## 2 Materials and methods

### 2.1 Participants

The sample size for this study was initially estimated using G*Power 3.1 software (Dusseldorf, Germany), with effect size (*ES*), α, and power (*1-β* error probability) set to 0.25, 0.05, and 0.95, respectively. The selected *ES* of 0.25 corresponds to a medium effect, as defined by Cohen (1988), and was based on previous biomechanical studies in individuals with FAI, which reported large *ES* in joint angles and GRF during landing tasks (Wang et al., 2024). Based on this estimation, the total sample size required was calculated to be 18. In this study, 20 male unilateral functional ankle instability individuals were selected, and both lower limbs underwent the drop landing task [Age (23.50 ± 0.76) years, Height (175.50 ± 5.37) cm, Weight (71.90 ± 7.93) kg; CAIT (Cumberland Ankle Instability Tool) score: unstable side (19.20 ± 2.38), stable side (28.50 ± 0.51), *p*< 0.001]. The study followed the Declaration of Helsinki and was approved by the Ethics Committee of Soochow University (SUDA20221005H03). All participants were fully informed about the purpose of the study and provided written informed consent. They were diagnosed with FAI based on established screening criteria and had a minimum of 3 years of regular sports training experience.

The inclusion and exclusion criteria for participants were as follows:

Inclusion Criteria:

I. CAIT score less than 24 on the unstable side (Donahue et al., 2011).
II. A CAIT score of 24 or higher for the stable side (Hiller et al., 2007).
III. No history of severe lower limb injuries, such as fractures or serious orthopedic injuries (Wu et al., 2022).
IV. At least one ankle sprain on the unstable side in the past year.

Exclusion criteria:

I. Bilateral ankle sprains (Wang et al., 2022).
II. Acute pathological symptoms in the lower limbs, such as acute fractures, cellulitis, and acute muscle strains, and so on.
III. History of lower limb fractures, surgeries (Kweon et al., 2022), hypertension, heart disease, or other conditions that may interfere with testing.
IV. CNS, vestibular disorders, or visual impairments that could affect balance (Wu et al., 2022).
V. Congenital deformities of the foot, ankle, knee, pelvis, or spine.
VI. Positive results on the anterior drawer test or talar tilt test (Kaminski et al., 1999).

### 2.2 Data collection

**FIGURE 1:**
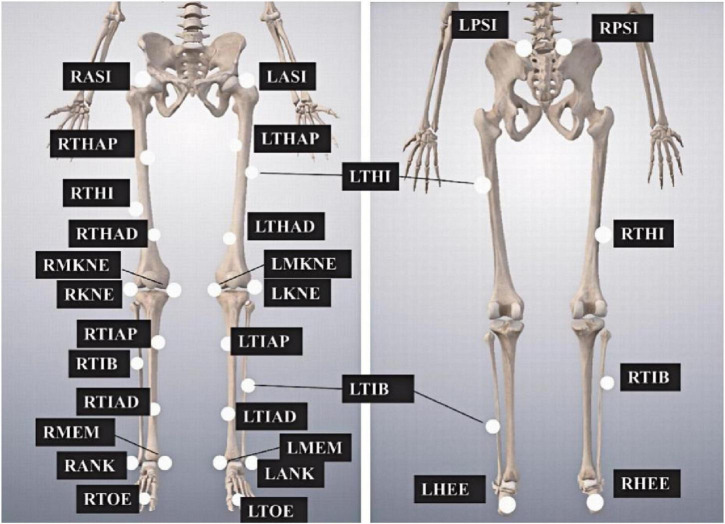
Markers placement on the lower limbs. The abbreviations are as follows, LASI and RASI, left and right anterior superior iliac spines, respectively; LTHAP and RTHAP, left and right thigh alignment upwards; LTHI and RTHI, left and right thigh; LTHAD and RTHAD, left and right thigh alignment downwards; LMKNE and RMKNE, left and right medial knee; LKNE and RKNE, left and right knee; LTIAP and RTIAP, left and right tibia alignment upwards; LTIB and RTIB, left and right tibia; LTIAD and RTIAD, left and right tibia alignment downwards; LMEM and RMEM, left and right medial malleoli; LANK and RANK, left and right ankle; LTOE and RTOE, left and right toe; LPSI and RPSI, left and right posterior superior iliac spines; LHEE and RHEE, left and right heel.

A Vicon motion capture system (Vicon Nexus v.1.5.1) equipped with eight infrared cameras (MX13, United Kingdom) and 28 reflective markers (14 mm in diameter) were used to collect the kinematic data at a sampling frequency of 100 Hz. The placement of markers on the lower limbs is shown in Figure 1. GRFs were recorded synchronously using a force plate (9287B, Kistler, Inc., Switzerland), operating at a sampling frequency of 1,000 Hz and connected to the motion capture system via an analog-to-digital converter.

### 2.3 Experimental procedure

By integrating slow, moderate, and fast tempo conditions (Kim et al., 2023; Kirkland et al., 2018; Rivera-Tello et al., 2023), this experimental design included four rhythm conditions (no rhythm, 60, 120, and 180 bpm) to investigate the effects of auditory rhythm on lower limb joint mechanics during single-leg drop landing in individuals with FAI. Before the experiment, participants changed into standardized athletic shoes (Feiyue, China), cotton socks, and experimental attire, and the laboratory temperature was maintained at a constant 23°C. Participants refrained from strenuous activity for 24 h before the experiment to prevent muscle fatigue. After completing a 5-min warm-up on a treadmill at 2.2 m/s, reflective markers were promptly placed by the same experienced experimenter within 3 min to preserve the effects of the warm-up. Before performing the drop landing task under rhythm conditions, a structured rhythm adaptation paradigm was implemented to enhance rhythmic adaptation effects. Specifically, the single-leg drop landing task began with the control condition (CC), which did not involve rhythmic stimuli and therefore did not require prior rhythm adaptation. The remaining three conditions (60, 120, and 180 bpm) were completed in a randomized order. During the period of rhythm adaptation, participants were seated at a distance of 55 cm from the computer screen (Lenovo Legion R7000, 15.6-inch, 144 Hz) and underwent a rhythm adaptation phase for 1-min firstly, using a metronome software app (Metronome, v15.2, Japan), which generated auditory beats at predefined tempos. To ensure that the rhythm variations reflected differences in tempo rather than beat strength, all rhythms were represented in terms of 1/4-note lengths. After that, the three rhythm conditions (60, 120, and 180 bpm) were converted into time inter-onset interval (IOI, ms) of 1,000 500, and 333.33 ms, respectively, using the formula (1).


(1) $$\mathrm{metronome}\left(M.M.,\mathrm{bpm}\right)=\frac{60000}{\mathrm{IOI}}\left(Repp,1994\right),$$

In this formula, 60,000 represents the total number of milliseconds in 1 min, IOI refers to the inter-onset interval in milliseconds, which is the time between two consecutive beats.

**FIGURE 2:**
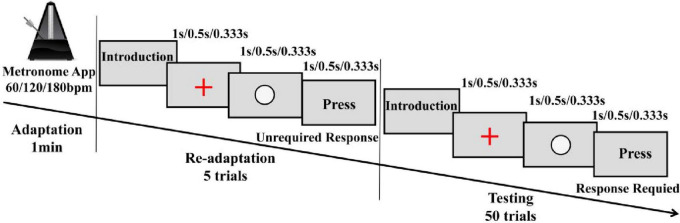
Rhythm adaptation paradigm.

These calculated IOIs were applied within the readaptation and test phases of rhythm adaptation paradigm, which designed using Python-based Psychopy software (v 2023.2) to objectively assess participants’ rhythm adaptation based on their response time errors. In the readaptation phase, participants were presented five times with three elements on the screen (a “+” symbol, a white circle, and a “Press” text)—which were displayed sequentially according to the rhythm (Figure 2), the IOI between them was identical to the target interval (Breska and Ivry, 2018). During this phase, participants did not need to respond but only familiarized themselves with the rhythmic structure. However, they were instructed to always pay close attention to the auditory rhythm cues. In the test phase, the elements displayed were the same, participants were instructed to respond by pressing the spacebar when the “Press” text appeared at rhythmically defined time points. This phase included 50 trials, and the timing of the keypress was recorded. The accuracy of the response was assessed based on the error between the participant’s keypress and the expected rhythm. A correct response was defined as a keypress occurring within ± 50 ms of the expected rhythm (Darabi and Svensson, 2021). Rhythm adaptation was considered successful if accuracy was at least 80% (Fuller and Fienup, 2018). If this threshold was not met, participants had to undergo re-adaptation, with a 120–s rest period provided between two adaptation attempts.

**FIGURE 3:**
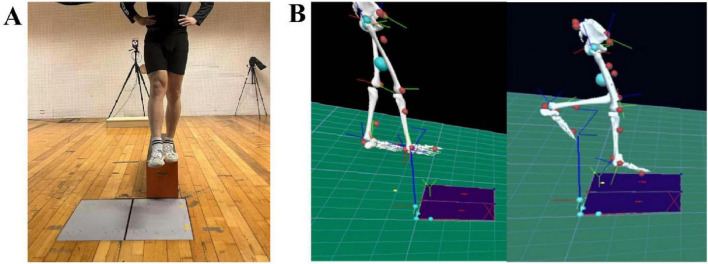
Preparation for the drop landing test **(A)** and lower limb model **(B)**. **(A)** represents the step-off motion during the drop landing movement, while **(B)** illustrates the pyCGM2-lowerLimb_CGM23 lower-limb model in Visual3D.

After successfully completing the rhythm adaptation task, participants moved on to the single-leg drop landing task. They stood on a 30 cm high platform with their hands on their hips (Figure 3A) to avoid interfering with the motion capture markers and to minimize the influence of arm movement. Participants in the rhythm conditions listened to the beats and independently determined when to step off the platform and land on their test leg, which was randomized between the left and right leg for each trial. For all conditions, the experimenter provided a verbal instruction (“Prepare, left/right foot, trial 1/2/3”) before each trial. The left/right foot cue was included because the experimenter was unaware of the unstable side, and the trial number helped track the three required valid trials for each condition. A trial was considered valid if the participant landed with the forefoot transitioning to the heel and maintained a stable single-leg stance for 3 s (Ardakani et al., 2019). Data collection began when the experimenter issued the command and ended once the participant stabilized the landing and exited the cushioning phase. Each participant collected three valid drop landings per condition, with a 1-min rest period between trials to minimize fatigue(Wernli et al., 2016). To enhance data reliability and reduce inter-trial variability, mean values from three valid drop landings per condition were calculated and used for subsequent analysis(Troester et al., 2018). No additional rest period was provided between different rhythm conditions, as participants naturally rested on their bodies during the rhythm adaptation paradigm task. All single-leg drop landing tests (2 sides: stable and unstable × 4 conditions: CC, 60, 120, 180 bpm) were completed within a single experimental day.

### 2.4 Experimental indicators

In this study, various GRF and joint kinetic variables were selected for analysis under different rhythm conditions during drop landing task. The GRF parameters included vertical ground reaction force (vGRF, N) at initial ground contact (IC) and peak vertical ground reaction force (PvGRF) were normalized to body weight (BW), and the time to peak vertical ground reaction force (T_PvGRF, ms) was also recorded. IC was defined as the moment when the vGRF first exceeded 10N (Christoforidou et al., 2017). The range of motion (RoM, °) for the hip, knee, and ankle joints was also measured in degrees. The RoM was calculated as the difference between the maximum and minimum joint angles recorded during the entire drop landing phase. The entire landing phase was defined from IC until the vGRF returned to 1 BW, indicating the end of the drop landing buffering process(Nauwelaerts and Aerts, 2006).

The pyCGM2-lowerLimb CGM23lower-limb model in Visual3D (C-Motion, United States, Figure 3B) was used to calculate joint kinetics. The joint torques (Nm/kg) at the PvGRF moment for the hip, knee, and ankle joints at sagittal plane were analyzed. Additionally, stiffness parameters were assessed, including lower limb stiffness (K_hip_, BW/m) and joint stiffness (K_joint_, dNm/kg/°).

To improve the numerical representation of joint stiffness, we expressed the stiffness values in deci-Newton meters per kilogram per degree (dNm/kg/°), where 1 dNm = 0.1 Nm. This unit adjustment allows for greater precision and more decimal places in the presentation of small stiffness values, without changing the underlying physical measurement. The symbol “+” denotes hip extension, knee extension, and ankle plantarflexion, while “-” denotes hip flexion, knee flexion, and ankle dorsiflexion. The lower limb stiffness was calculated using Eq. (2), while joint stiffness was derived from Eq. (3):


(2) K=legPvGRF/ΔL(Yin et al., 2020),

(3) K=jointΔM/RoM(Farley et al., 1998),

where PvGRF represents the peak vertical GRF, which refers to the maximum vGRF. ΔL represents the vertical displacement of the hip joint center. ΔM represents the change in joint torques, and RoM represents the change in joint angle. The K_joint_ model was based on the ratio of net muscle moment change to joint angular displacement in the sagittal plane during the ground-contact phase. This approach assumes that the joint behaves approximately as a torsional spring when the time difference between peak torque and peak angular displacement is minimal (typically less than 10% of the movement cycle). Under such conditions, the joint stiffness metric provides a reasonable mechanical approximation of dynamic joint behavior.

### 2.5 Statistical analysis

Data analysis was performed using SPSS 26.0 and Excel 2021 software, with a significance level set at α = 0.05. All data were presented as mean ± standard deviation (M ± SD). First, the Shapiro-Wilk test was used to assess whether the data followed a normal distribution. If the data were normally distributed, two-way repeated measures analysis of variance (rmANOVA) was used to analyze the main effects, interaction effects, and simple effects of side (stable side vs. unstable side) and rhythm conditions (no rhythm, 60, 120, and 180 bpm) on the variables during the drop landing task. For variables that were not normally distributed, non-parametric tests such as the Friedman test were used to examine overall differences, followed by post hoc comparisons using the Wilcoxon signed-rank test with Bonferroni correction to control for multiple comparisons.

## 3 Results

### 3.1 GRF

**FIGURE 4:**
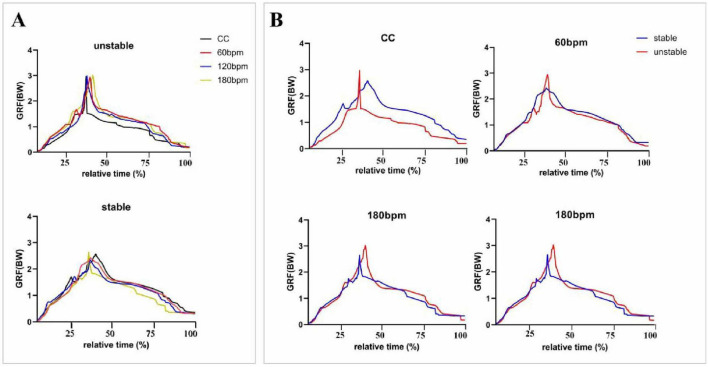
Vertical ground reaction forces (vGRFs) during the entire drop landing phase. The vGRF (N) data were normalized by dividing by body weight (BW), and the results were expressed as multiples of BW. CC, the condition of no rhythm (control condition). The x-axis represents relative time (%), indicating the progression of vGRF from the IC moment to the PvGRF moment and the end of the cushioning phase. **(A)** Presents an overall comparison of vGRF trends across all rhythmic conditions for both the stable and unstable sides, while **(B)** depicts the vGRF trends separately for the stable and unstable sides under each rhythmic condition.

The diagram of vGRF during the entire drop landing phase is shown in Figure 4. Significant main effect of side was observed for both the vGRF at IC moment and PvGRF (*p*< 0.05). Specifically, the vGRF at IC moment was higher on the unstable side than on the stable side (*F*_1,38_ = 5.857, *p* = 0.020, *η_*p*_*^2^ = 0.134), and the PvGRF was also higher on the unstable side (*F*_1,38_ = 6.943, *p* = 0.012, *η_*p*_*^2^ = 0.154). No significant differences were found for T_PvGRF (*p*> 0.05), as detailed in Table 1.

**TABLE 1:** ANOVA results on GRFs and joint torques.

Parameter		Factor	Df	*F*-value	p-value	η_*p*_^2^
GRFs	vGRF	Side	1.38	5.857	0.020*	0.134
		Condition	3.114	0.562	0.618	0.015
		Side × condition	3.114	0.366	0.749	0.010
	PvGRF	Side	1.38	6.943	0.012*	0.154
		Condition	3.114	1.358	0.271	0.102
		Side × condition	3.114	0.113	0.952	0.009
	T_PvGRF	Side	1.38	0.016	0.899	0.005
		Condition	3.114	0.136	0.938	0.011
		Side × condition	3.114	0.466	0.708	0.037
Joint torques at IC moment	Hip	Side	1.38	2.373	0.132	0.059
		Condition	3.114	3.903	0.015*	0.093
		Side × condition	3.114	2.931	0.044*	0.072
	Knee	Side	1.38	12.686	0.001*	0.250
		Condition	3.114	7.360	<0.001*	0.162
		Side × condition	3.114	12.494	<0.001*	0.247
	Ankle	Side	1.38	32.512	<0.001*	0.461
		Condition	3.114	6.652	0.001*	0.357
		Side × condition	3.114	9.729	<0.001*	0.448
Joint torques at PvGRF moment	Hip	Side	1.38	20.908	<0.001*	0.355
		Condition	3.114	1.050	0.370	0.027
		Side × condition	3.114	14.238	<0.001*	0.273
	Knee	Side	1.38	54.577	<0.001*	0.590
		Condition	3.114	4.211	0.010*	0.100
		Side × condition	3.114	9.047	<0.001*	0.192
	Ankle	Side	1.38	0.730	0.398	0.019
		Condition	3.114	18.305	<0.001*	0.325
		Side × condition	3.114	0.683	0.547	0.018

*p<0.05. vGRF, vertical ground reaction force at initial ground contact (IC) moment; PvGRF, the peak vertical ground reaction force; T_PvGRF, time to peak vertical ground reaction force.

### 3.2 Lower limb joint torques

#### 3.2.1 Joint torques at IC moment

As shown in Table 1, at the IC moment, the hip joint torque was significantly influenced by the interaction effects of rhythm condition and side, and by the main effect of rhythm condition (*p*< 0.05). Simple effects analysis revealed that the hip extension torque on the unstable side under CC was greater than under 180 bpm (*p* = 0.010). Under 120 bpm condition, the hip extension torque on the stable side was greater than on the unstable side (*F*_1,38_ = 6.242, *p* = 0.017, *η_*p*_*^2^ = 0.141), and the 180 bpm condition also showed a greater hip extension torque on the stable side (*F*_1,38_ = 4.239, *p* = 0.046, *η_*p*_*^2^ = 0.100).

As for the knee joint torque, there was a significant interaction between the effects of rhythm condition and side (*p*< 0.001), as well as the main effects of rhythm condition (*p*< 0.001) and side (*p* = 0.001). Simple effects analysis revealed that, the knee extension torque on the stable side, under CC was greater than under both 60 and 180 bpm (*p*< 0.001). On the stable side, the knee extension torque under 120 bpm was greater than under both 60 bpm (*p* = 0.005) and 180 bpm (*p*< 0.001). On the unstable side, the knee extension torque at 60 bpm was greater than under CC (*p* = 0.023), 120 bpm (*p* = 0.008), and 180 bpm (*p*< 0.001). Under CC and 120 bpm, the knee extension torque on the stable side was greater than on the unstable side (*F*_1,38_ = 17.314, *p*< 0.001, *η_*p*_*^2^ = 0.313; *F*_1,38_ = 14.863, *p*<0.001, *η*_*p*_^2^ = 0.281, respectively), whereas at 60 bpm, the knee extension torque on the unstable side was greater than on the stable side (*F*_1,38_ = 8.220, *p* = 0.007, *η*_*p*_^2^ = 0.178).

**FIGURE 5:**
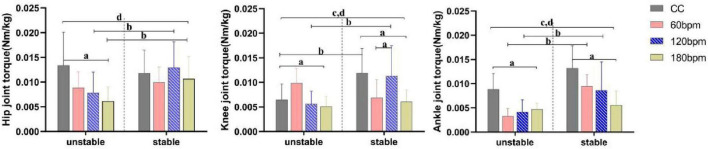
Joint torques at initial ground contact (IC) moment. a,b Indicate a statistically significant interaction effect between side and rhythm condition (*p* <0.05). a Indicates a significant simple effect of rhythm; b Indicates a significant simple effect of side. c Indicates a statistically significant main effect of side (*p* < 0.05). d Indicates a statistically significant main effect of rhythm condition (*p* < 0.05).

Ankle joint torque was significantly influenced by the interaction effects of rhythm condition and side (*p*<0.001), as well as the main effects of rhythm condition (*p* = 0.001) and side (*p*<0.001; Table 1). Specifically, the ankle plantarflexion torque on the stable side under 180 bpm was smaller than under CC (*p* = 0.001) and 60 bpm (*p* = 0.007). On the unstable side, the ankle plantarflexion torque under CC was greater than under both 60 (*p* = 0.001) and 120 bpm (*p* = 0.08).,The ankle plantarflexion torque on the stable side under 60 bpm was greater than the unstable side (*F*_1,38_ = 55.231, *p*< 0.001, *η_*p*_*^2^ = 0.592), and the ankle plantarflexion torque on the stable side under 120 bpm was greater than on the unstable side (*F*_1,38_ = 7.365, *p* = 0.010, *η_*p*_*^2^ = 0.162) (Figure 5).

#### 3.2.2 Joint torques at PvGRF moment

At the PvGRF moment, hip joint torque was significantly influenced by the interaction effects of rhythm condition and side, as well as by the main effect of side (*p*< 0.001, Table 1). On the stable side, the hip extension torque under CC was greater than under the 180 bpm condition (*p* = 0.037). On the unstable side, the hip extension torque under CC was smaller than under both the 120 (*p* = 0.025) and 180 bpm (*p* = 0.003). Additionally, the hip extension torque on the unstable side at 60 bpm was smaller than at 120 (*p* = 0.001) and 180 bpm (*p*< 0.001). On the other hand, the hip extension torque on the unstable side was greater than the stable side under both 120 and 180 bpm (*F*_1,38_ = 20.175, *p*< 0.001,*η_*p*_*^2^ = = 0.347; *F*_1,38_ = 44.232, *p*< 0.001,*η*_*p*_^2^ = = 0.538, respectively).

Knee joint torque was significantly influenced by the interaction effects of rhythm condition and side (*p*<0.001), as well as by the main effects of condition (*p* = 0.010) and side (*p*< 0.001, Table 1). Specifically, the knee extension torque on the stable side under 180 bpm was smaller than under CC (*p* = 0.026), 60 bpm (*p* = 0.016) and 120 bpm conditions (*p*< 0.001). The knee extension torque on the unstable side under CC was smaller than under the 60 (*p* = 0.013) and 180 bpm (*p* = 0.035) conditions. Regardless of the rhythm condition, the knee extension torque on the stable side was always greater than on the unstable side (CC, *F*_1,38_ = 32.487, *p*<0.001, *η_*p*_*^2^ = 0.461; 60 bpm, *F*_1,38_ = 11.932, *p* = 0.001,*η_*p*_*^2^ = 0.239; 120 bpm, *F*_1,38_ = 44.965, *p*<0.001,*η_*p*_*^2^ = 0.542; 180 bpm, *F*_1,38_ = 4.365, *p* = 0.043, *η*_*p*_^2^ = 0.103).

**FIGURE 6:**
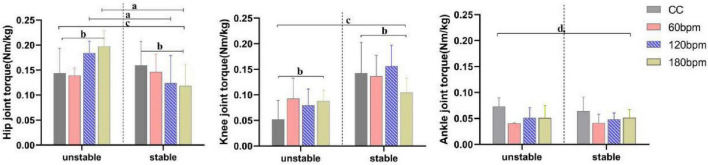
Joint torques at PvGRF moment. a,b Indicate a statistically significant interaction effect between side and rhythm condition (*p* < 0.05). a Indicates a significant simple effect of rhythm (*p* < 0.05). b Indicates a significant simple effect of side (*p* < 0.05). c Indicates a statistically significant main effect of side (*p* < 0.05). d Indicates a statistically significant main effect of rhythm condition (*p* < 0.05).

Ankle joint torque was significantly influenced by the main effect of rhythm condition (*p*<0.001, Table 1). The ankle plantarflexion torque under CC was greater than under all three rhythm conditions, and the ankle plantarflexion torque at 60 bpm was the lowest (*p*< 0.001). No significant main effect of side (*F*_1,38_ = 0.730, *p* = 0.398,*η_*p*_*^2^ = 0.019) or interaction effect (*F*_3,114_ = 0.683, *p* = 0.547,*η_*p*_*^2^ = = 0.018) was observed for ankle joint torque (Figure 6).

### 3.3 RoM

Table 2 presents the descriptive statistics of joint RoM under different rhythmic conditions for both limbs, and the results of the ANOVA are summarized in Table 3. The RoM in the hip joint and knee joint was significantly affected by rhythm condition (*F*_3,114_ = 8.282, *p*<0.001, *η_*p*_*^2^ = 0.179; *F*_3,114_ = 4.468, *p* = 0.008, *η_*p*_*^2^ = 0.105, respectively). The hip RoM under CC was smaller than under the rhythmic conditions, and the hip RoM under 180 bpm was higher than other conditions. The knee RoM under CC was the highest. No significant differences were observed for ankle RoM (*p* > 0.05).

**TABLE 2:** The Hip, knee and ankle RoM across four auditory rhythm conditions for both limbs (M ± SD).

Parameter		Hip (°)	Knee (°)	Ankle (°)
CC	Unstable	19.82 ± 5.93	16.46 ± 5.90	21.61 ± 5.42
	Stable	19.16 ± 4.43	17.17 ± 6.70	19.78 ± 8.13
60 bpm	Unstable	21.00 ± 4.75	15.41 ± 5.06	19.44 ± 6.53
	Stable	23.63 ± 4.14	15.38 ± 5.79	18.51 ± 7.23
120 bpm	Unstable	20.64 ± 3.55	15.24 ± 4.97	20.16 ± 7.60
	Stable	24.56 ± 3.98	13.80 ± 5.69	16.02 ± 6.89
180 bpm	Unstable	23.36 ± 4.71	14.98 ± 5.46	18.92 ± 6.97
	Stable	26.29 ± 4.53	13.84 ± 5.84	16.72 ± 6.73

RoM, range of motion. The hip, knee, and ankle joints RoM were measured in degrees (°).

**TABLE 3:** ANOVA results on RoM and stiffness.

Parameter		Factor	Df	*F*-value	*p*-value	η_*p*_^2^
RoM	Hip	Side	1.38	0.424	0.519	0.011
		Condition	3.114	8.282	<0.001*	0.179
		Side × condition	3.114	1.709	0.173	0.043
	Knee	Side	1.38	0.093	0.762	0.002
		Condition	3.114	4.468	0.008*	0.105
		Side × condtio	3.114	0.889	0.436	0.023
	Ankle	Side	1.38	1.257	0.269	0.032
		Condition	3.114	1.661	0.204	0.118
		Side × condition	3.114	1.039	0.387	0.080
Stiffness	K_leg_	Side	1.38	2.487	0.123	0.061
		Condition	3.114	6.909	0.001*	0.365
		Side × condition	3.114	0.432	0.732	0.035
	K_hip_	Side	1.37	4.261	0.046*	0.103
		Condition	3.35	2.447	0.080	0.173
		Side × condition	3.111	5.798	0.003*	0.332
	K_knee_	Side	1.38	1.679	0.203	0.042
		Condition	3.114	0.439	0.727	0.035
		Side × condition	3.114	0.305	0.821	0.025
	K_ankle_	Side	1.38	8.729	0.005*	0.187
		Condition	3.114	10.989	<0.001*	0.478
		Side × condition	3.114	0.810	0.497	0.063

*p < 0.05. RoM, range of motion; K_leg_, lower limb stiffness; K_hip_, hip joint stiffness; K_knee_, knee joint stiffness; K_ankle_: ankle joint stiffness.

### 3.4 Stiffness

K_hip_ was significantly influenced by rhythm condition (*F*_3,36_ = 6.909, *p* = 0.001, *η_*p*_*^2^ = 0.365). The stiffness at 60 bpm was significantly lower than under CC, 120 and 180 bpm conditions. K_hip_ was significantly influenced by the interaction of rhythm condition and side (*F*_3,111_ = 5.798, *p* = 0.003, *η_*p*_*^2^ = 0.332), as well as by the main effect of side (*F*_1,37_ = 4.261, *p* = 0.046,*η_*p*_*^2^ = 0.103). Specifically, the K_hip_ on the stable side under 60 bpm was greater than under CC (*p* = 0.003), 120 (*p* = 0.04) and 180 bpm (*p* = 0.006). At 60 bpm, the K_hip_ on the stable side was greater than on the unstable side (*F*_1,37_ = 16.586, *p*< 0.001, *η_*p*_*^2^ = 0.310). No significant differences were observed for K_knee_ (*p*> 0.05). K_ankle_ was significantly influenced by the main effects of rhythm condition and side (*F*_3,36_ = 10.986, *p*<0.001,*η_*p*_*^2^ = 0.478; *F*_1,38_ = 8.729, *p* = 0.005, η_*p*_^2^ = 0.187, respectively). The stiffness on the unstable side was greater than on the stable side, with the K_ankle_ at 180 bpm being the highest and the stiffness at 60 bpm being the lowest, as the ANOVA results detailed in Table 3 and the descriptive statistics detailed in Table 4.

**TABLE 4:** The lower limb stiffness and joints stiffness across four auditory rhythm conditions for both limbs (M ± SD).

Parameter		K_leg_	K_hip_	K_knee_	K_ankle_
CC	Unstable	24.65 ± 9.78	1.17 ± 0.30	0.10 ± 0.02	0.13 ± 0.01
	Stable	20.30 ± 4.96	1.28 ± 0.34	0.19 ± 0.04	0.09 ± 0.01
60 bpm	Unstable	20.48 ± 4.21	0.77 ± 0.25	0.12 ± 0.02	0.08 ± 0.01
	Stable	16.88 ± 4.05	2.01 ± 0.65	0.16 ± 0.02	0.05 ± 0.01
120 bpm	Unstable	23.02 ± 7.95	0.71 ± 0.27	0.09 ± 0.02	0.08 ± 0.01
	Stable	21.56 ± 7.97	1.02 ± 0.31	0.21 ± 0.05	0.05 ± 0.01
180 bpm	Unstable	23.79 ± 8.66	0.79 ± 0.33	0.09 ± 0.01	0.18 ± 0.01
	Stable	22.69 ± 7.67	1.04 ± 0.38	0.15 ± 0.02	0.11 ± 0.01

K_hip_, lower limb stiffness; measured in BW/m; K_hip_, hip joint stiffness; K_knee_, knee joint stiffness; and K_ankle_, ankle joint stiffness, measured in dNm/kg/°.

## 4 Discussion

This study investigates the effects of auditory rhythm adaptation on lower limb joint mechanics in FAI individuals. The results confirmed our hypotheses, rhythmic auditory cues modulated range of motion and torques, and different rhythm conditions elicited distinct bilateral mechanical responses during single-leg drop landing. Compared to the control condition, plantarflexion torque at the PvGRF moment was significantly reduced under any rhythmic conditions, particularly at 60 bpm, while the 120 bpm elicited increased hip and knee extension torques. Meanwhile, the stable side showed greater RoM. In contrast, the unstable side consistently exhibited reduced hip and knee torques and restricted hip RoM across all conditions, reflecting impaired neuromuscular control.

### 4.1 The effect of different auditory rhythms adaptation

At the PvGRF moment, the plantarflexion torque under CC was higher than any rhythmic conditions. A higher plantarflexion moment may reflect greater mechanical demands on the ankle joint. This increased torque may indicate compensatory strategies or inefficient load absorption mechanisms, both of which have been associated with elevated susceptibility to reinjury in unstable ankles (Delahunt et al., 2006; Gribble et al., 2013). Therefore, compared to conditions with rhythmic stimulation, the absence of rhythm may lead to suboptimal joint loading patterns and compromised joint protection. Auditory rhythms can influence motor output by entraining motor-related neural oscillations, thereby aligning cortical activity with rhythmic timing and facilitating more temporally coordinated movement (Clements-Cortes and Bartel, 2018). This synchronization often referred to as entrainment (Braunlich et al., 2019). In the context of motor control, such entrainment may serve as a mechanism through which external auditory cues modulate joint-level movement patterns. Interestingly, plantarflexion torque at 60 bpm was the lowest on both the stable and unstable side. One possible explanation is that at 60 bpm, the ankle joint may not have had sufficient time to generate an effective shock absorption response, leading to reduced torque output. Although slower rhythm theoretically offers a longer preparatory window, the reduced K_hip_ and torque outputs suggest that the entrainment effect was suboptimal. This finding aligns with the idea that suboptimal rhythmic entrainment may interfere with anticipatory postural adjustments (APAs)—the preemptive neuromuscular activations critical for preparing the body for landing. Ineffective APAs at this slower rhythm could result in delayed or inefficient muscle coordination, contributing to altered joint mechanics during complex motor tasks. Instead, the system may have redistributed neuromuscular control to proximal joints, minimizing direct ankle load but requiring longer postural adjustment times.

In contrast, under 120 bpm, the hip, knee, and ankle torques on the stable side were higher than those on the unstable side at IC moment, suggesting a torque redistribution strategy in which the stable limb assumes a greater mechanical role in impact attenuation at IC moment. The effectiveness of 120 bpm may be attributed to its alignment with natural human physiological rhythm (Iwanaga, 1995), enhancing sensory-motor synchronization and the coupling of perception and action. Studies have found that rhythms in the range of 76-122 bpm induce stronger neural activity in the auditory cortex, posterior cingulate cortex, and inferior parietal lobule (Liu et al., 2018). Styns et al. (2007) also found that rhythms between 106 and 130 bpm elicited a favorable synchronization with human body movements. These findings further substantiate the significant role of a 120 bpm rhythm in enhancing athletic performance and motor control. This entrainment effect at 120 bpm may offer unique benefits by enhancing temporal predictability and facilitating rhythm-driven motor timing. Meanwhile, at the PvGRF moment, the hip extension torque on the unstable side exceeded that of the stable side, may reflect a neuromuscular adaptation in response to impaired ankle function, whereby joint loading is shifted proximally to reduce mechanical stress on the compromised distal segments. Such a hip-dominant control strategy likely contributes to postural stabilization and reflects an internal protective mechanism to maintain functional integrity during high-impact tasks. These findings highlight the role of auditory rhythm adaptation in modulating joint-specific motor strategies. The observed improvements in joint torque profiles and load redistribution suggest that rhythm-based interventions may serve as a promising non-invasive approach to enhance joint coordination and motor control in individuals with FAI.

However, under 180 bpm, although the stable side demonstrated the greatest hip RoM, it simultaneously exhibited reduced RoM in both the knee and ankle joints, along with the lowest ankle plantarflexion torque. These results suggest that faster rhythms may increase the motor execution demands. This suggests that while rhythm adaptation can enhance control, excessively rapid tempos may impose constraints on inter-joint coordination and functional stability.

### 4.2 Differential joint mechanics in the bilateral lower limbs of FAI individuals

At the IC moment, the vGRF and the PvGRF were higher on the unstable side, suggesting that the unstable side may exhibit deficits in neuromuscular control, thus increasing the risk of injury during drop landing.

The study also revealed that, under both the 120 and 180 bpm, the stable side exhibited higher hip and knee extension torques at IC moment, along with a greater hip RoM, compared to the unstable side. These rhythms fall within the range of human-preferred movement rhythms and are commonly used in sports and rehabilitation settings to guide coordination and movement timing. The observed increase in joint extension at IC moment may serve as a preparatory strategy to absorb impact forces, with the extended posture facilitating elastic deformation and energy dissipation during drop landing (Zhang et al., 2000).

On the other hand, the unstable side under CC exhibited lower hip and knee extension torques at PvGRF moment and smaller hip RoM compared to rhythmic conditions. However, under 120 bpm, the hip extension torque on the unstable side increased, higher than the stable side, indicating an enhanced proximal joint contribution. This change may reflect the modulatory role of auditory rhythm adaptation in enhancing neuromuscular coordination, particularly through the temporal synchronization of sensory input and motor output (Thaut et al., 1999; Todd and Lee, 2015). Prior studies have shown that auditory-motor synchronization improves movement accuracy, enhances preparatory motor activity, and supports postural regulation during dynamic tasks (Repp, 2005; Schaefer, 2014). Thus, the mechanism of drop landing is not merely mechanical, involving shock absorption and energy conversion, but also includes neurophysiological modulation of motor timing and postural strategies. This integrated response may be especially beneficial for individuals with sensorimotor deficits, such as those with FAI.

According to Fernández-Sotos et al. (2016), rhythmic units consist of one or more pulses in a sustained pattern, serving as temporal cues that can guide movement. These rhythmic pulses may influence motor control by enhancing sensorimotor integration. Damm et al. (2020) demonstrated that auditory rhythm can couple with motor instructions, enhancing the connection between perceptual and motor regions. Consistent with these findings, our results suggest that auditory rhythm adaptation may enhance neuromuscular control on the unstable side of FAI individuals, potentially improving motor performance, and the hip joint played a key role in stability and control. Notably, the increased engagement of proximal joints observed under the 120 bpm condition may represent a general motor adaptation strategy that supports efficient torque redistribution, optimized load management.

The hip, knee, and ankle joints together form an integrated kinetic chain in the lower limb, and these joints work in concert to perform movement tasks. When dysfunction occurs in one joint, the body adapts by modifying its movement strategy, relying on adjacent or distal joints to compensate for the impaired joint (Lee et al., 2018). In this study, the unstable side exhibited a greater hip extension moment under CC and at the PvGRF moment. This suggests that, in the presence of ankle instability, individuals subconsciously rely more on the hip joint to buffer the impact forces during drop landing. However, this reliance on the hip joint may represent a passive compensatory movement pattern. Proprioception is crucial for neuromuscular control. Ankle proprioceptors relay joint position, movement status, and muscle force information to the brain, which then processes this feedback to precisely regulate neuromuscular activity (Fernandes et al., 2000). On the unstable side, impaired proprioceptive input may cause “*neurological input blockage*” (Pereira et al., 2022), impairing the CNS ability to respond effectively (Linford et al., 2006). As a result, individuals may become passively dependent on the hip joint, reducing the ankle’s ability to absorb shock during drop landing (Hubbard and Wikstrom, 2010). Following an ankle injury, the body may adapt by developing movement patterns that limit load on both the injured and uninjured ankle.

K_joint_ refers to the joint’s resistance to deformation under external force, indicating the joint’s stability and flexibility. While a stiffer joint inherently requires greater force to achieve the same deformation, this does not equate to improved stability or reduced flexibility in isolation. Instead, stiffness represents a trade-off between energy absorption and structural rigidity during dynamic tasks like drop landing. During impact absorption, higher K_joint_ enhances force dissipation capacity, thereby reducing peak stress on articular surfaces and soft tissues. Our findings revealed that K_hip_ at 60 bpm was highest on the stable limb, aligning with its role in load-bearing stability. Conversely, the unstable limb exhibited paradoxically higher K_ankle_ at 180 bpm, despite the ankle’s functional requirement for greater flexibility. This counterintuitive result suggests a compensatory mechanism that reduced ankle mobility, evidenced by RoM decrements at 180 bpm, may force the joint into a stiffer mechanical state to maintain positional control, potentially increasing injury risk through altered load distribution.

### 4.3 Limitations and future directions

It is important to acknowledge that our estimation of K_joint_ is based on a simplified biomechanical model that considers only external joint torque and joint angular displacement, and does not incorporate muscle properties such as viscosity, activation timing, or viscoelastic behavior. Consequently, the model may omit critical neuromuscular factors that influence joint stiffness under real-life dynamic conditions. Future studies should consider integrating electromyography (EMG) and muscle ultrasound data to capture the active muscular contributions to joint stabilization and to more comprehensively assess joint protection strategies during high-impact tasks. Second, the drop landing task may not fully capture delayed motor responses that could occur under 60 bpm. Future research could explore the biomechanical adaptations of FAI individuals when performing rhythmic entrainment tasks under slow-tempo constraints using gait or stepping tasks.

Finally, given that rhythmic cues can support motor coordination and sensory function (Hsu et al., 2021), incorporating rhythmic elements into conventional training, such as balance exercises, strength training, and proprioceptive neuromuscular facilitation (PNF) techniques, may potentially enhance timing control, movement consistency, and the efficiency of motor learning. Such combinations yield additive or synergistic benefits could also be systematically investigated.

## 5 Conclusion

Rhythmic auditory adaptation influences lower limb joint mechanics and modulates motor control strategies in individuals with FAI during single-leg drop landing. Specifically, auditory stimulation at 120 bpm elicited a proximal-dominant torque distribution pattern on the stable limb at IC moment, characterized by increased hip and knee extension torques. Meanwhile, torque redistribution and increased hip joint involvement on the unstable side at PvGRF moment reflects compensatory adaptations aimed at mitigating stress on the unstable ankle. These findings highlight the potential of rhythm-based interventions, particularly 120 bpm, to optimize anticipatory motor strategies and reduce reinjury risk in FAI individuals through non–invasive modulation of sensorimotor coordination.

## Data Availability

The original contributions presented in the study are included in the article/supplementary material, further inquiries can be directed to the corresponding authors.
